# Use of geographic information systems web mapping application to support active case search to guide public health and social measures in the context of COVID-19 in Zimbabwe: a preliminary report to guide replication of methods in similar resource settings

**DOI:** 10.11604/pamj.2021.38.159.27143

**Published:** 2021-02-12

**Authors:** Isah Mohammed Bello, Thandekile Ntombikayise Moyo, Manes Munyanyi, Godwin Ubong Akpan, Irene Isibor, Lincoln Charimari Sunganai, Abubakar Sadiq Umar, Ravi Shankar Santhana Gopala Krishnan, Kebba Touray, Maxwell Rupfutse, Portia Manangazira, Alex Gasasira Ntale, Daniel Fussum, Pascal Mkanda

**Affiliations:** 1 World Health Organization, Inter-Country Support Team office for East and Southern Africa, Harare, Zimbabwe,; 2 World Health Organization, African Regional Office, Brazzaville, Congo,; 3 World Health Organization, Harare, Zimbabwe,; 4 World Health Organization, Headquarters, Geneva, Switzerland,; 5 Ministry of Health and Child Care, Harare, Zimbabwe

**Keywords:** GIS, COVID19, Zimbabwe, active case search, geo-spatial analysis

## Abstract

**Introduction:**

the new coronavirus (COVID-19) that emerged from Wuhan, Hubei Province of China in December 2019, causing severe acute respiratory syndrome (SARS) has fast spread across the entire globe, with most countries struggling to slow and reduce the spread of the virus through rapid screening, testing, isolation, case management, contact tracing, implementing social distancing and lockdowns. This has been shown to be a major factor in countries that have been successful in containing COVID-19 transmission. Early detection of cases is important, and the use of geospatial technology can support to detect and easily identify potential hotspots that will require timely response. The use of spatial analysis with geographic information systems (GIS) had proved to be effective in providing timely and effective solutions in supporting epidemic response and pandemics over the years. It has developed and evolved rapidly with a complete technological tool for representing data, model construction, visualization and platform construction among others.

**Methods:**

we conducted a geospatial analysis to develop a web mapping application using ArcMap and ArcGIS online to guide and support active case search of potential COVID-19 cases, within 500m radius of COVID-19 confirmed cases to improve detection and testing of suspected cases.

**Results:**

the web mapping application tool guides the active case search teams in the field, with clear boundaries on the houses to be visited within 500-meter radius of confirmed positive cases, to conduct active case search of all cases of severe acute respiratory illnesses (SARI), acute respiratory illnesses (ARI), pneumonia etc, to detect and test for COVID-19 towards containing the pandemic.

**Conclusion:**

the use of GIS and spatial statistical tools have become an important and valuable tool in decision-making and, more importantly, guiding health care professional and other stakeholders in the response being carried out in a more coherent and easy manner. It has proven to be effective in supporting the active case search process to rapidly detect, test and isolate cases during the process, towards containing the COVID-19 pandemic.

## Introduction

A new virus (SARS-CoV-2) emerged in December 2019 from Wuhan, Hubei province of China, causing severe acute respiratory syndrome (SARS) and spreading fast to all continents of the world. On 30 January 2020, the World Health Organization (WHO) declared that the new SARS-CoV-2 (COVID-19) outbreak constitutes a Public Health Emergency of International Concern (PHEIC), with over 5,05,885 confirmed cases and 333,446 deaths in about 216 countries as of May 23 2020, negatively affecting social and economic development of most countries [1]. The pandemic is directly threatening all the achievements gained in realization of the united nations sustainable development goals (SDGs), which aimed to ensure healthy lives and promote well-being for all people at all ages by addressing social, economic and environmental issues from 2015 to 2030 [2]. Following the declaration of COVID-19 as a PHEIC, the WHO outlined several levels of intervention with the main goal for countries to be able to control COVID-19 outbreak by slowing down the transmission of the virus and preventing morbidity and mortality [1]. Each country is required to use the epidemiology of the virus and based on local context, to guide implementation of strategies and measures [3]. World Health Organization had published strategy update [4], which is intended to guide countries at both levels to tailor their response based on local context by ensuring faster and more effective processes of finding all suspected cases of COVID-19, testing, isolation, treatment of the confirmed cases and subsequently contact identification/listing and follow up to contain the spread of the virus [5]. One critical component of these approaches is case finding, which can be implemented through an effective active, passive and event-based surveillance. Active surveillance is the principal approach to rapidly identify cases consists of close supervision and systematic collection of timely information on vital signs and symptoms from suspected individuals and their contacts [6].

It involves searching for all suspected cases in all health facilities and the community including cases severe acute respiratory illnesses (SARI), acute respiratory illnesses (ARI), pneumonia etc. Active surveillance had proven to be a very effective mechanism for searching cases and had been used extensively by different health programs to interrupt the spread of diseases. Use of active case finding as a strategy for improving detection of Ebola virus disease (EVD) cases in Nimba County, Liberia [7]. Use of active case finding (ACF) and enhanced case finding (ECF) in detecting Tuberculosis (TB) cases by health care system towards increasing detection [8]. Use of active surveillance-response system in enhancing early case detection, diagnosis and treatment in an effort towards reducing/eliminating severe ulcers and its related disabilities in Ghana West municipality of Ghana [9]. While there are lots of evidence in the use of GIS in supporting surveillance (active and passive), its use in guiding intervention in the recent fight against COVID-19 has been limited. The use of paper-based system in collecting data had been challenged with issues related to delay in searching of records, limited or no backup system available, loss of records, extra storage space for keeping and backup etc [10], hence the recent shift towards a more systematic computerized systems. In recent times, the use geographic information system (GIS) had proven to be an effective tool in the field of public health due to its potential for providing real time information to facilitate appropriate evidence-based intervention measures. Health professionals are using conventional mapping, and geographic information systems (GIS) as a tool for tracking diseases and combating contagion [11]. Geographic information system methods to improve case detection through improving community health for SARS outbreak [12], its wide application in public health research [13], and in mapping and visualizing disease distribution towards understanding diversities of diseases and spatial patterns in health science [14].

Considering the magnitude of the COVID-19 pandemic and its negative effect on socio-economic indices, there is the need to track the coronavirus pandemic using different platforms, ranging from online/mobile GIS and mapping application/dashboard [11]. While there are lots of evidence in the use of GIS in supporting surveillance (active and passive) on COVID-19 in developed countries (reference), its use is limited in the developing countries. The COVID-19 pandemic in Zimbabwe present unique challenges that will require the use of innovative approaches as most of the cases were predominantly in two (2) major cities of the country (Harare and Bulawayo). The country had adopted a very aggressive laboratory testing strategy that is targeting all patients meeting the case definition of suspected cases of COVID-19, all travellers arriving from countries with community transmission of COVID-19, patients with influenza-like illnesses (ILI), patients with pneumonia, and all community deaths amongst others. However, identifying the current level of local or community transmission, hot spots within the country requires strategies that will effectively guide outbreak managers in the right direction. The response team therefore, decided to conduct a risk assessment, through the use of house-to-house active case search in the community with positive cases, aimed at actively searching for all suspected cases, testing and isolation, while at the same time delivering messages to address knowledge, attitude and perception at the household and community levels. Hence, GIS technology was deployed to guide and support the process. In this paper, we developed a web mapping application using the ArcGIS online tool to guide teams in the field on the exact houses to be visited based on the 500-meter radius surrounding each positive confirmed case. This application was built using ArcGIS web mapping application to display the 500m radius boundary to locate, identify, monitor and track the exact houses to be visited by the teams during the assessment process.

## Methods

**Figure 1:**
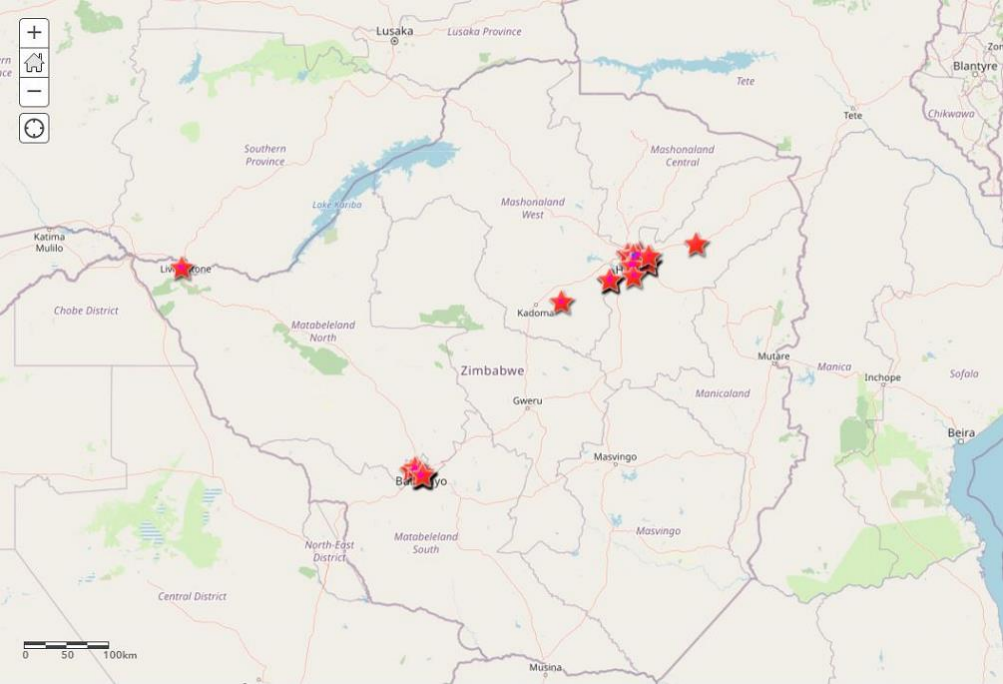
mapped COVID-19 positive cases using coordinates location

**Figure 2:**
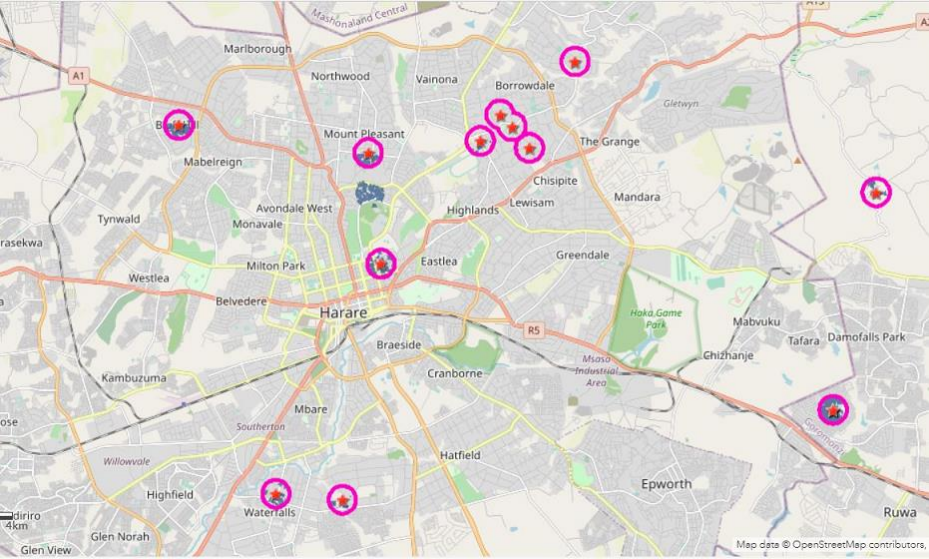
500m radius buffer created around confirmed COVID-19 cases demarcating the boundary and showing the households to be covered

This is a cross-sectional study design that tries to document the methodology and processes of using the geo-spatial analysis to guide the selection of houses and locations for surveys and active case search. The ArcGIS Pro and ArcGIS online were used to develop the web mapping application. The line list of confirmed positive cases, with details of the exact location of the cases, was used (longitude and latitude) to display the location of the cases on the map. The cases were uploaded on the ArcGIS online platform, showing the exact location of the confirmed cases (Figure 1), and a buffer of a 500-meter radius was created around each of the confirmed positive case, which delineates the exact houses to be covered by each team for the assessment (Figure 2). The Zimbabwe building footprints were also added to the buffer to help estimate the total number of houses to be targeted in each buffer, as well as support in planning for logistics and number of teams to be deployed to each location (real-time marathon was adopted for the location with incomplete building footprints) to ensure all buffers have building identified.

## Results

**Figure 3:**
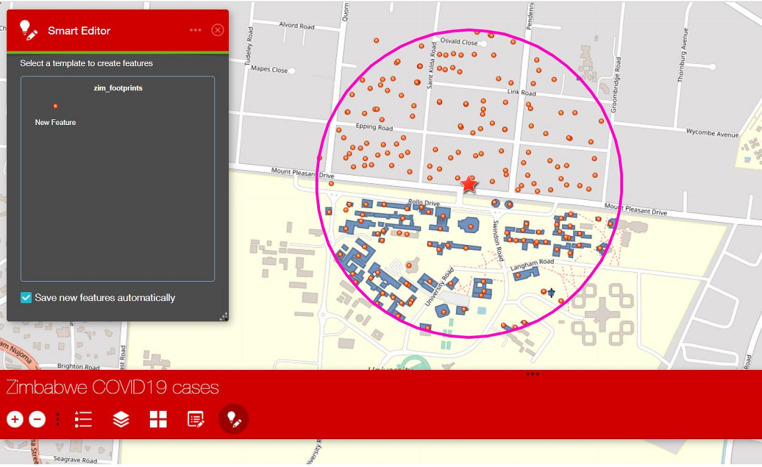
web mapping application with building footprints and editing options, with allows users to locate and update households to be visited within the buffer

**Figure 4:**
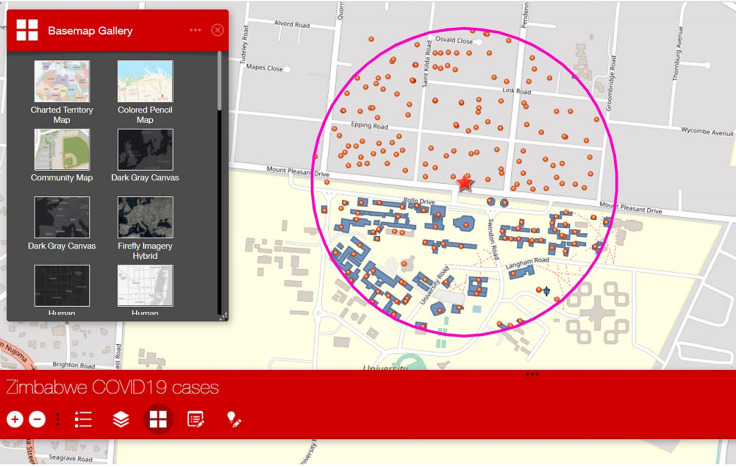
web mapping application with base map options, with allows users to use and clearly identify boundaries and households to be visited within the buffer

A web mapping application was then developed to be used in guiding team members in the field and deployed on tablets/smartphones. However, the building footprints were not complete for all cities of Zimbabwe and hence, we designed the application to include editing options where users could co-locate buildings that are within each buffer using the smart editor to update all building that is within each buffer area (Figure 3). The team is composed of three (3) people, two (2) house to house personnel deployed from the health facility, and another personnel who is experienced in social research to collect information on knowledge, attitudes, practices regarding COVID-19 preventive measures. A multi-layered supervisory structure (1^st^, 2^nd^, 3^rd^ and 4^th^) are assigned to each team to consolidate the information collected by the house-to-house team and use it to target individuals/families/communities for additional assessment and laboratory testing, as well as review all the data from the campaign and use it to fine-tune interventions at community and district level [15]. Each rapid response team was equipped with a smartphone with the web mapping application deployed in it, and also papers for documenting and listing all cases that met the criteria to be swabbed and tested; the teams also conducted active case search within health facilities (HFs) in the area (if available) guided by the application in both real-time and offline (including private clinics) to sensitize health workers. The social mobilizers in the team will direct all clients (community members with flu-like symptoms) to the strategic location of the rapid response team (RRT) team, where clinicians will screen the clients and collect specimens from suspected cases. For each household visited by the team, they captured the coordinates location of the household, which is later compared to the actual household expected to be visited by the team to detect missed households. A base map gallery was added to the web mapping application to allow users the options to choose different base maps (open streets) towards providing a clearer view of the street names in these locations for guidance, the map is interactive and the team could zoom in and out as well as use additional features that are embedded in the application (Figure 4).

## Discussion

**Implementation and rollout plan:** the solution was first piloted in Harare city, which was the epicenter of the outbreak, and was then subsequently rolled out to other provinces of the country with reported confirmed cases. The team had previously worked with android applications like Open Data Kit (ODK), district health information software (DHIS2) etc, and currently using the ODK application for case investigation and contact tracing. The training was conducted for all members of the rapid response teams (RRT) from provinces with confirmed cases, where the application was presented, and guidance was given on its usage. The team members went for field practical to demonstrate the usage of the application and familiarize themselves with all the features that came with the application. A dashboard was created that compares the expected number of houses to be visited versus the actual houses visited to detect missed houses and provide feedback to the team on household coverage on daily basis for each buffer area by location.

**Resource implications:** the development and deployment of this application would not require additional cost to the response, because all the required infrastructure for the platform had been provided by World Health Organization (WHO) to support COVID-19 response. Additionally, there are already existing tablets and smartphones provided by WHO to support the response, which can be utilised for this activity. Furthermore, the rapid response team has a health information officer attached to each of the teams, who can use the application deployed on tablets to guide and direct the teams on the exact houses to be visited for the assessment. World Health Organization has provided two (2) additional technical officers geographic information systems and monitoring and experience (GIS and M&E) to support the process. The Ministry of Health and Child Care (MOHCC) has also provided additional staff from the health information unit to support the process including data collation and analysis at the end of the assessment. However, some of the challenges that may hinder successful implementation includes internet availability in some locations of the country for the real-time update, cost of data bundles, as well as an outage of battery power for the tablets in the field.

## Conclusion

The use of GIS technologies had proved to be an effective tool in improving data sharing and real-time information to support critical decision-making and can be used as an effective tool for supporting non-pharmaceutical mitigation measures in the fight against COVID-19 pandemic response. The use of this tool in the COVID-19 response in Zimbabwe will significantly guide and support the active case search team to easily identify houses to be visited by the team in order to promptly detect, test and isolate cases towards control and eventual containment of the pandemic, thereby reducing the risk of transmission of the virus.

### What is known about this topic

- Geographic information system (GIS) and geo-spatial analysis tools are evolving and widely used to support decision making, currently been deployed to support in the fight against COVID-19;
- The use of non-pharmaceutical measures is adopted by countries to slow down and break the chain of COVID-19 transmission;
- Active case finding had proven to be an effective tool used for case search, and is being used by different countries to detect COVID-19 cases in the community.

### What this study adds

- The use of geographic information system and spatial analysis tools provided an insight and had demonstrated the scalability and speed, coupled with geospatial thinking during COVID-19 response;
- The use of active case search in communities with confirmed COVID-19 can be integrated with health education and sensitization to increase awareness of COVID-19 pandemic;
- Use of non-pharmaceutical intervention was employed by different countries towards breaking the chain of transmission in the fight against COVID-19.
